# Context-dependent roles of B cells during intestinal helminth infection

**DOI:** 10.1371/journal.pntd.0009340

**Published:** 2021-05-13

**Authors:** Aidil Zaini, Kim L. Good-Jacobson, Colby Zaph

**Affiliations:** 1 Infection and Immunity Program, Monash Biomedicine Discovery Institute, Monash University, Clayton, Victoria, Australia; 2 Department of Biochemistry and Molecular Biology, Monash University, Clayton, Victoria, Australia; University of Utah, UNITED STATES

## Abstract

The current approaches to reduce the burden of chronic helminth infections in endemic areas are adequate sanitation and periodic administration of deworming drugs. Yet, resistance against some deworming drugs and reinfection can still rapidly occur even after treatment. A vaccine against helminths would be an effective solution at preventing reinfection. However, vaccines against helminth parasites have yet to be successfully developed. While T helper cells and innate lymphoid cells have been established as important components of the protective type 2 response, the roles of B cells and antibodies remain the most controversial. Here, we review the roles of B cells during intestinal helminth infection. We discuss the potential factors that contribute to the context-specific roles for B cells in protection against diverse intestinal helminth parasite species, using evidence from well-defined murine model systems. Understanding the precise roles of B cells during resistance and susceptibility to helminth infection may offer a new perspective of type 2 protective immunity.

## Introduction

Intestinal helminths infect over 1.5 billion people worldwide [1,2]. The most common types of human intestinal helminths are roundworm (*Ascaris lumbricoides)* infecting 819 million people, whipworm (*Trichuris trichiura)* infecting 465 million people, and hookworm (*Necator americanus*) infecting 439 million people [3,4]. These parasites can establish a chronic infection and inhabit the host intestine for years, leading to a wide range of comorbidities such as malnutrition, anemia, and impaired cognitive development [5]. Due to the high prevalence of these parasites in tropical and subtropical regions, intestinal helminth infection is an alarming concern for human health and socioeconomic stability of affected populations. The current treatment for intestinal helminth infection in high-risk areas is periodic administration of deworming drugs that has been shown to be relatively effective at reducing the intensity of infection and therefore protecting populations at risk from morbidity. However, multiple treatments of the drug fail to prevent reinfection [6]. Although rodent models have significantly advanced our understanding of both protective and ineffective immune responses to helminth infection, the long-term immunological interventions such as anthelmintic vaccines have yet to be successfully developed [7]. It has been well established that the main drivers of an effective anti-helminth immune response are CD4^+^ T helper (Th) cells. However, the roles for B cells in protective responses during helminth infection are arguably the most variable and highly context dependent, leading to many interesting hypotheses for how B cells function during these responses. Understanding the requirements that dictate why and how B cells are important during helminth infection may pave the way for new long-term immunological interventions. Here, we summarise the findings for the myriad roles of B cells in type 2 immunity using evidence from a variety of murine intestinal helminth models and discuss the factors that determine their requirement for protection against helminths.

## Immune responses to helminths

**Fig 1:**
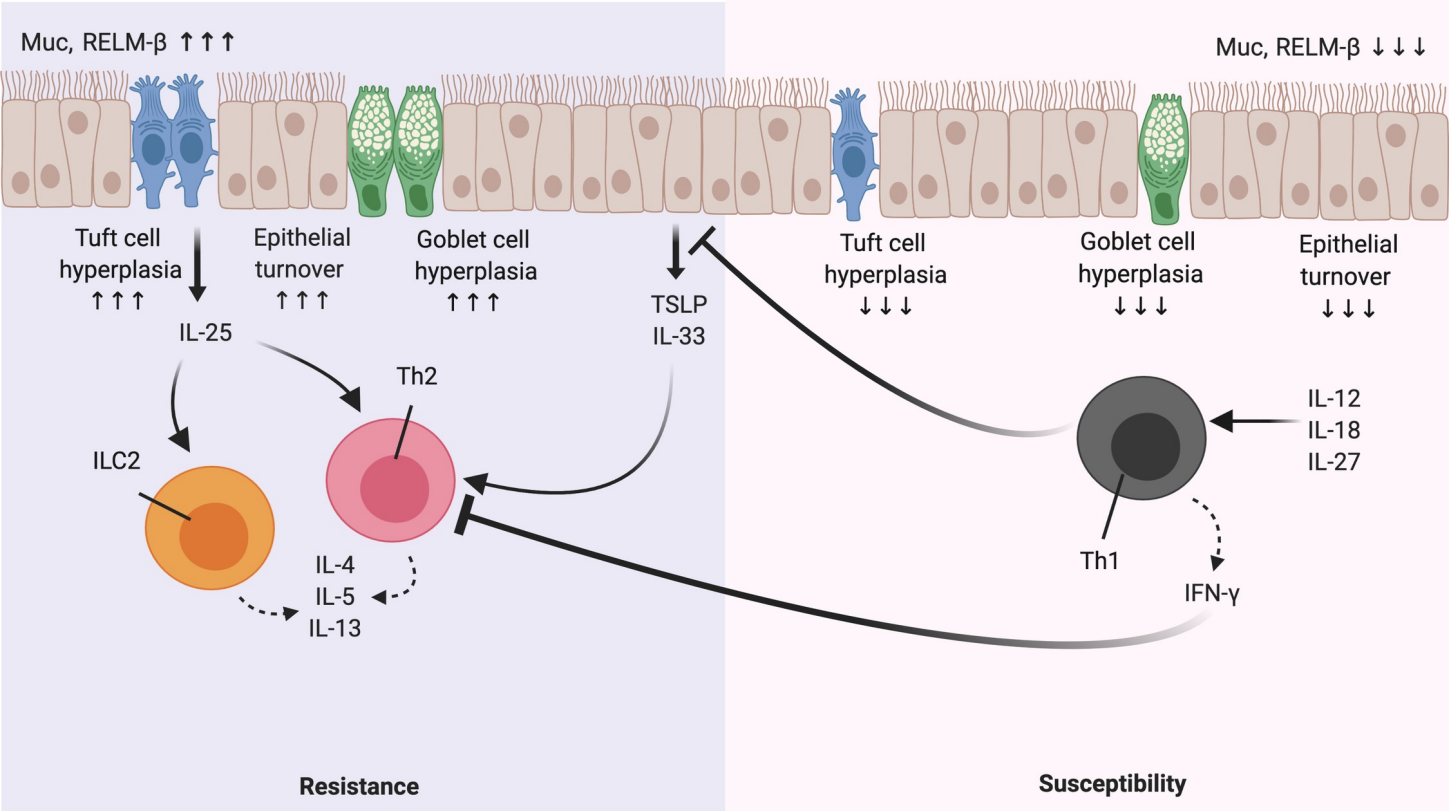
A paradigm of Th1/Th2 response during helminth infection. During a Th2 cell-biased response, epithelial cell-derived cytokines such as TSLP and IL-33, in addition to tuft cell-derived IL-25, collectively result in the activation of type 2 response through Th2 cells and ILC2s. Once activated, these cells produce the type 2 cytokines IL-4, IL-5, and IL13 that in turn activate effector mechanisms for worm expulsion such as goblet cell hyperplasia, increased mucus production (Muc), muscle contractility, and production of RELM-β. In contrast, a Th1-biased response results in susceptibility. Activated IL-12-, IL-18-, and IL-27-induced Th1 cells (under some circumstances) robustly produce IFN-γ, which synergistically represses the type 2 response. IFN-γ, interferon gamma; ILC2, innate lymphoid cell 2; Muc, mucin; RELM-β, resistin-like molecules β; TSLP, thymic stromal lymphopoietin.

Protective immunity to helminth infection is thought to be mediated by a type 2 immune response that is primarily governed by Th2 cells [8]. A protective Th2 cell-biased response is typically initiated at the infection site [9]. In mesenteric lymph nodes (MLNs), activated dendritic cells (DCs) present helminth-derived antigens to polarise naive Th cells into Th2 cells via major histocompatibility complex class II–T cell receptor (MHCII–TCR) interactions and specific costimulatory signals [10]. Once activated, Th2 cells are capable of producing type 2 cytokines such as IL-4, IL-5, IL-9, and IL-13 that are collectively responsible for the effector mechanisms of worm expulsion [9] (Fig 1). Consistent with strong evidence from murine models of helminth infection, ex vivo analysis of blood from individuals with ascariasis demonstrates an inverse association between Th2 cytokines and infection intensity [11], suggesting that acquired Th2 response is a defining feature of resistance to helminths [12]. The Th2 cell-derived cytokines amplify the Th2 cell-biased response through a positive feedback loop [13]. The type 2 protective immunity elicited by a wide range of innate and adaptive cells during helminth infection are collectively important for worm expulsion; however, the response can also be variable and influenced by the types of worms, distinct host–parasite interactions, and the stages of infection. The effector mechanisms for worm expulsion typically include increased epithelial cell turnover, goblet cell hyperplasia, increased mucus secretion (mucins Muc2 and Muc5ac), muscle contractility, and elevated production of resistin-like molecule β (RELM-β) [9] (Fig 1), which have been thoroughly reviewed elsewhere [9,14].

In contrast, Th1 cell-skewed responses with high levels of interferon gamma (IFN-γ) production are often associated with chronic intestinal helminth infections, particularly in murine models such as in response to the whipworm *Trichuris muris*. However, the IFN-γ-mediated Th1 response is not the defining feature of susceptibility to *Trichinella spiralis* [15]. The roles of Th1 response and how Th1-independent responses drive susceptibility in response to intestinal helminths have been extensively reviewed elsewhere [16]. In susceptible mice that induce a Th1-biased response, specific helminth-derived antigens drive the production of type 1 cytokines such as IL-12 and IFN-γ [17] (Fig 1). Through the production of IL-12, migratory CD103^+^ DCs suppress Th2 cell development, leading to the proliferation of Th1 cells in MLNs [18]. In addition to IL-12, IL-18 and IL-27 can also promote the induction of Th1 responses under some circumstances [19–22]. Once Th1 cells are expanded, they migrate toward the lamina propria and produce the cytokine IFN-γ, resulting in a diminished Th2 cell response [23].

One of the main roles of Th2 cells is to facilitate B cell responses to infection [24–26]. While it is well established that a Th2-biased response underpins resistance to helminth infection, the protective roles for B cells are not as clear-cut. Different murine models of helminth infections, primary versus secondary infection (i.e., recall responses), developmental stages of helminth, experimental settings, and strains of mice investigated in the studies are all factors likely to contribute to such discrepancies. In this review, we will further discuss the roles and regulation of B cells during helminth infection.

## Type 2 germinal center (GC) responses: A component of anti-helminth immunity?

**Fig 2:**
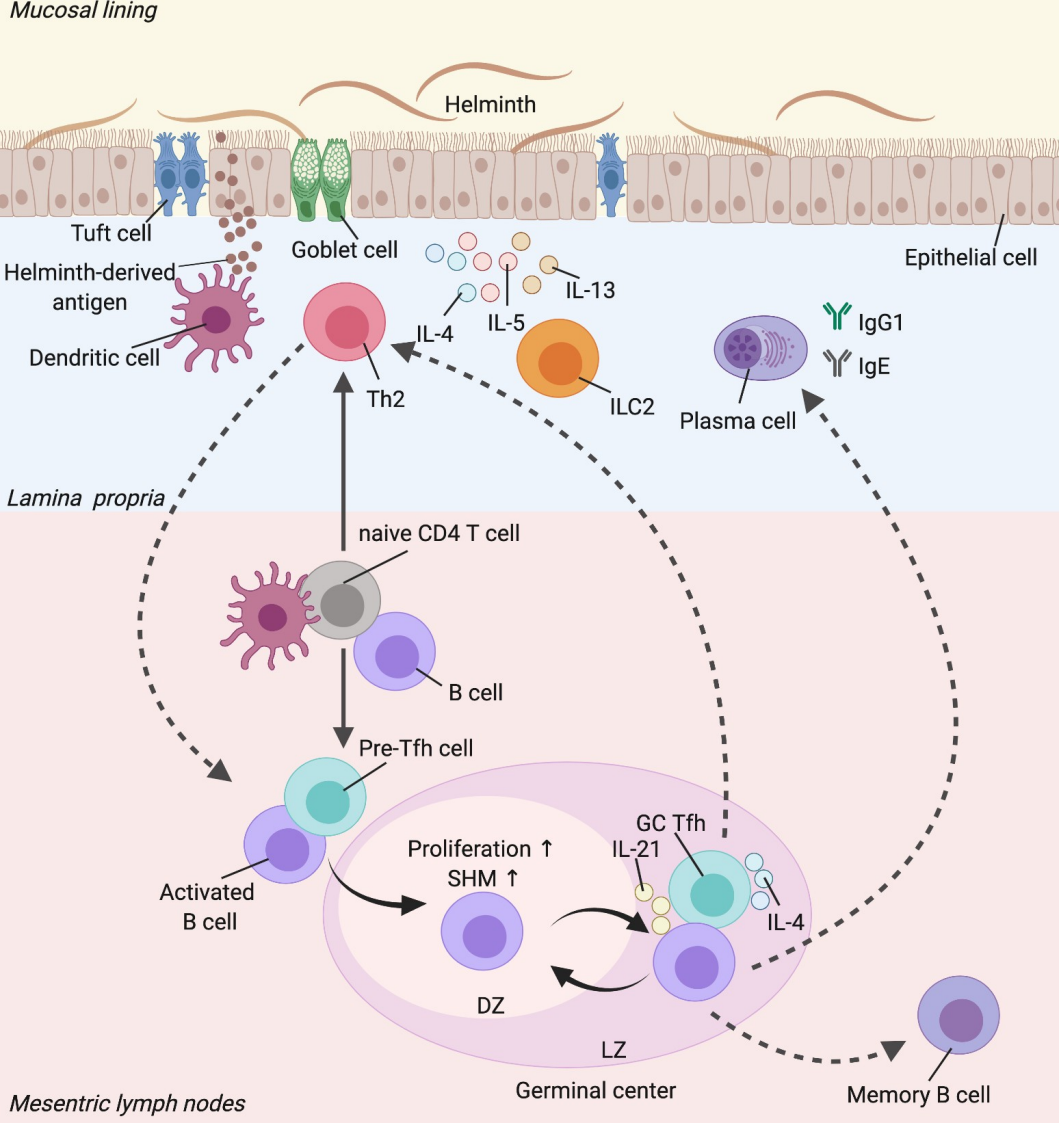
The protective roles of a GC-Tfh response during helminth infection. During early stages of helminth infection, DCs in the lamina propria process the parasite-derived antigen, which then migrate to the MLNs. In MLNs, the DCs alongside B cells interact with naive CD4 T cells that result in Th2 and Tfh cell differentiation. In the presence of specific signals, Th2 cells are then localised to the effector sites and are predominantly responsible for the effector type 2-mediated worm expulsion response. On the other hand, Tfh cells interact with activated B cells for the formation of GC reactions in the MLNs. In the DZ, the B cells undergo further proliferation and SHM, resulting in higher affinity of B cell receptors. The B cells then migrate to the LZ and are selected by Tfh cells via specific interactions and Tfh-derived cytokines such as IL-4 and IL-21, to return to the DZ. Such a process occurs in an iterative fashion in order to produce an effective antibody repertoire such as IgG1 and IgE. The GC-Tfh cells can also differentiate into effector Th2 cells and vice versa. The GC-derived B cells can develop into long-lived memory B cells (Image created with Biorender.com). DC, dendritic cell; DZ, dark zone; GC, germinal center; ILC2, innate lymphoid cell 2; LZ, light zone; MLN, mesenteric lymph node; SHM, somatic hypermutation.

B cells are a crucial component of adaptive immune responses, primarily governing humoral immunity through an effective antibody repertoire and B cell memory. There are 2 pathways of Th cell-dependent B cell activation which are responsible for intermediate and persistent humoral immunity. One is the extrafollicular response, which occurs outside lymphoid follicles of the secondary lymphoid organs and is mainly essential for generating plasmablasts as an important source of early protective antibodies [27,28]. Due to help signals provided by Tfh cells such as via CD40–CD40L interactions, activated B cells can enter the GC pathway [29]. The protective roles of helminth-specific B cells have not been well established due to varying and sometimes contradicting findings between different intestinal helminth species. The primary mechanism of how B cells are involved in protective immunity to helminths has been proposed to be through antibody production. Antibodies can either activate the type 2-associated innate cells to promote worm expulsion or directly bind to the parasites, which will be discussed in-depth later. Typically, long-lived and high-affinity antibodies are an output of GCs [30]. However, whether the GC response can mediate protective immunity to helminths by generating high-affinity antibodies that either directly or indirectly promote the effector mechanisms of worm expulsion or whether GCs can promote the differentiation of protective Th2 cells remains unclear (Fig 2).

### GC dynamics during helminth infection

Upon an encounter with a foreign antigen, B cells migrate across distinct microanatomical compartments and interact with both immune and stromal cells and eventually establish GCs. Once fully established, GCs generate a sustained phase of antibody diversification and affinity maturation. Currently, there is limited data suggesting that GCs are required for protection against helminths. However, a number of studies have demonstrated robust GC formation during *Litomosoides sigmodontis* [31], *Heligmosomoides polygyrus* [32–35], and *Nippostrongylus brasiliensis* [36–38] infection. The immune response that develops following helminth infection is highly dynamic and changes as the parasite develops, from the detection of the parasite by the innate immune system to the induction of adaptive responses, ultimately leading to parasite expulsion [9]. Although it is clear that the type 2 immune response promotes resistance to infection, it remains enigmatic as to what specific types of B cell responses are associated with early and the late stages of worm expulsion. Since Th cell number typically peaks weeks earlier than the GC response [35], it is possible that GCs might be particularly responsible for the late stages of worm expulsion or fecundity. For example, Th cells are the dominant lymphocyte that drives the immune response at day 12 postinfection of *H*. *polygyrus* [35]. However, at day 21 postinfection, the numbers of CD4 T cells start to decrease, leaving the persistent B cells, specifically within the B cell follicles as the dominant lymphocyte that drives the immune response in the MLNs [35]. Furthermore, the dissociation of lymphotoxin-α (LT)-mediated B cell follicles results in impaired worm expulsion [35]. It is thus tempting to speculate that Th2 cells and GC formation progressively develop until a point at which Th2 cells functionally decline, while the GC remains persistent until the infection is resolved. Therefore, it is likely that Th2 cells contribute to the anti-helminth response during the early stages of infection, while the GC reaction might be partly responsible for worm expulsion or fecundity during late stages of infection. This hypothesis however remains to be proven.

### Regulation of the GC response by Tfh cells

Tfh cells and GC B cells depend on each other to develop and function, via a wide range of molecular interactions that are central to the development of humoral immunity. Over the years, Tfh cells have been recognised as a major source of IL-4 in the lymph nodes (LNs) during the type 2 immune response. Although Th2 cells also produce IL-4, the roles of IL-4 production by Th2 cells are different compared with Tfh cells. For example, Th2 cell differentiation is dependent on IL-4 in an autocrine manner through a positive feedback loop [39,40]. Unlike Th2 cells, IL-4 is not required for Tfh cell development and functions [38,41–43]. However, IL-4 is indispensable for IgE/IgG1 class switching in activated B cells and is a regulator of the GC response. Th2 cells are commonly found at both the infection sites and in LNs. On the other hand, IL-4-producing Tfh cells are predominantly localised in close proximity to or within the B cell follicles of reactive LNs [38,41–43].

The requirement of IL-4 for protective responses is highly variable between different helminth infection models, which can also depend on the genetic background of the mice. For example, IL-4 is required for worm expulsion of *T*. *spiralis* only in C57BL/6 but not BALB/c mice [44]. Similarly, only *T*. *muris*-infected C57BL/6 but not BALB/c mice are susceptible to the infection in the absence of IL-4 [45]. Interestingly, in contrast to BALB/c male mice, IL-4-deficient females are resistant to *T*. *muris* infection, which is due to IL-13-mediated worm expulsion [45,46]. In addition, IL-4 is not required for worm expulsion following *N*. *brasiliensis* infection [47,48]. The extent to which IL-4 production by Tfh cells plays a role in IL-4-dependent protection against helminths however remains elusive.

T follicular regulatory (Tfr) cells are a distinct subset of the FOXP3^+^ T regulatory (Treg) cell population expressing Tfh signature markers such as CXCR5 and PD-1, whose primary function is thought to be a negative regulator of Tfh cell functions and thus is important for tuning the overall GC reaction [49–51]. While it is known that helminth infections such as *H*. *polygyrus* [52] and schistosomiasis in humans [53] induce the generation of Tfr cells, their roles in Th2-dependent parasite clearance are not examined in these studies. In Th2-dependent allergic responses, both suppressive and helper roles via IL-10 production to modulate IgE responses by Tfr cells have been identified; however, these roles are also dependent on the type of allergen (e.g., house dust mite versus peanut) [54,55]. Future studies aimed at dissecting the roles of Tfr cells in a helminth model of infection are required as it is difficult to extrapolate their roles from allergy models.

Tfh cells develop as the infection progresses and their IL-4 and IL-21 production regulates different aspects of the GC after soluble extract of *Schistosoma mansoni* egg-induced type 2 immunization [56] and *N*. *brasiliensis* infection [36]. Recently, it has been shown that a rare subpopulation of Tfh cells produces IL-13 to regulate high-affinity IgE responses during allergy but not following helminth infection [57]. IL-4 and IL-21 production by Tfh cells synergistically promotes the GC response during helminth infection. In particular, IL-21 production by Tfh cells is required for promoting somatic hypermutation (SHM) of GC B cells [36]. Furthermore, IL-21 is required for plasma cell differentiation but not the anti-helminth GC response, which is associated with successful worm expulsion during recall responses following *H*. *polygyrus* infection [58]. Interestingly, IL-21-deficient mice displayed an exaggerated response of the GC and Tfh cells [58]. Perhaps, IL-21 signaling is a limiting factor for the development of the type 2-associated GC B and Tfh cells during helminth infection. Conversely, IL-4 seems to be predominantly critical for the generation of GC-derived plasma cells, as well as their IgG1 and IgE production [36]. IL-4 production by Tfh cells is acquired during the associated stages of infection that parallels GC development, either during helminth [36,41] or viral infection [59]. In contrast, IL-21-producing Tfh cells appear to be more dominant during the early stages of GC development [36]. The dynamic production of IL-4 and IL-21 by Tfh cells may thus influence the outcome of protection against helminths.

Tfh cells can also express T-BET and produce IFN-γ although at lower levels than Th1 cells [60–62], highlighting the versatility of Tfh cell function. Tfh cells that produce IFN-γ can be referred to as Th1-like Tfh cells and have been implicated in regulating IgG2 class switch recombination in mice [63] and long-term maintenance of neutralizing antibodies [64]. Unlike viral infection, the studies on the roles of Th1-like Tfh cells during helminth infection are lacking as helminths typically induce a strong Th2 response. In C57BL/6 infected with a low dose of *T*. *muris* eggs (approximately 30), a Th1-biased immune response is induced and the mice fail to expel the worms [17]. In contrast, C57BL/6 mice given a high dose of *T*. *muris* eggs (approximately 150 to 200) develop a strong Th2-biased response, promoting worm expulsion. This dichotomy is not observed in Th2-prone BALB/c mice. The use of a low- and high-dose *T*. *muris* infection is a great tool that may help to elucidate the importance of Tfh and GC B cells in both the type 1 and 2 immune responses within the same helminth system and how regulation of Tfh-GC plays a role in protection against helminths.

### Functional plasticity of Tfh cells

A study by Zaretsky and colleagues [65] showed that Tfh cells develop from the same lineage of Th2 cells following type 2 immunization, suggesting that they both may have originated from the same precursor and that IL-4-producing Tfh cell differentiation from Th2 cells relies on the presence of GC B cells. In *H*. *polygyrus*-infected mice, intrinsic deletion of BCL6 in B cells impairs not only GC formation but also IL-4-producing Tfh cell differentiation [43]. In the context of recall Th2 responses induced by immunization, Tfh cell differentiation from memory T cells and their IL-4 expression occur through the interactions with the memory B cells that are likely to be derived from the GC during primary responses [56]. These interactions appear to be mutually important for both Tfh and B cells. It is thus possible that one of the pathways for the GC to indirectly exert protection against the parasites is through regulation of Th2-like Tfh cells during primary and recall responses following helminth infection (Fig 2). In a non-helminth Th2 cell response induced by house dust mite allergen, antigen-specific B cells prime the full differentiation of IL-4-expressing GC-Tfh cells that are the precursors of effector Th2 cells after rechallenge, highlighting an important plasticity that exists between Tfh and Th2 cells [66]. Such plasticity of Tfh cells that requires B cells also suggests GCs are a crucial reservoir of cells that regulate the development and regulation of Th2-like Tfh cells.

## Regulation of Th2 responses by B cells

B cells influence the magnitude of Th1/Th2 cell responses through their ability to produce effector cytokines, present antigens, express costimulatory molecules to enhance Th2 cell polarization, as well as their potential to interact with many other members of the type 2 immune responses including ILC2s. Two lineages of B cells include B1 and B2 cell subsets that are classified based on their unique development, cell surface marker expression, and immunoglobulin repertoire. For example, unlike bone marrow-derived B2 cells, B1 cells develop during fetal development [67,68]. Despite having a less diverse immunoglobulin repertoire than B2 cells [69], B1 cells have been thought to be partly essential for immunity against helminths by regulating Th2 responses during *L*. *sigmodontis* infection [70] and filariasis [71], with the evidence pointing to protective roles of B1 cells at supporting Th2 responses in other helminth infections is still lacking. It is also unclear how they would support Th2 responses as their activation is primarily via a Th-independent manner. The roles of B1 cells and their production of natural antibodies and IgM that results in either an exacerbation of or protection during parasitic infections, including helminths have been recently well documented elsewhere [72].

The absence of B cells in μMT^−/−^ mice on a C57BL/6 background favours a Th1-biased response by promoting IFN-γ production and thus resulting in susceptibility to *T*. *muris* infection [17]. Upon B cell transfer, μMT^−/−^ mice were able to expel the worms, suggesting B cells are important mediators of type 2 immune responses. However, CD4 T cell reconstitution into mice lacking both T and B cells (SCID) on a C57BL/6 background was sufficient to confer protection by targeting the larvae [73]. It is important to note that different strains of mice may dictate the roles of B cells in mediating Th2 response during helminth infection. For example, both *T*. *muris*-infected C57BL/6 and BALB/c mice are able to expel the worms; however, the former strain mounts mixed Th1/Th2 responses while the latter develops a strong Th2 cell response. A recent study confirmed that B cells are only able to enhance Th2 cell polarization during *T*. *muris* infection in a mixed Th1/Th2 environment [74]. The administration of monoclonal antibodies against IFN-γ [74] or IL-12 [17] into μMT^−/−^ mice on a C57BL/6 background was able to restore resistance by promoting Th2 cell responses. In support of these findings, the depletion of B cells via anti-CD20 treatment in *T*. *muris*-infected C57BL/6 but not BALB/c mice results in susceptibility [74]. These findings suggest B cells promote Th2 cell development and protection against helminths in a mixed Th1/Th2 environment.

### Antibody-independent functions of B cells: Enhancing Th2 responses

B cells are highly versatile due to their abilities to act as antigen-presenting cells (APCs) and provide costimulatory signals that are necessary for cell fate decisions of CD4 T cells, specifically Th2 cells [75]. Failure of B cells to interact with cognate CD4 T cells in the absence of B cell-specific MHC II expression results in an impaired Th2 cell response during primary and recall responses to *H*. *polygyrus* [76], as well as during Th2-biased immunization with antigens in alum [77]. More recently, a study demonstrates that IL-4Rα-expressing B cells are required for mediating effective immunity to secondary *N*. *brasiliensis* infection, which is associated with their abilities to interact with CD4 T cells as APCs for optimal Th2 cell response [78]. In addition to directly promoting Th2 cell polarization by priming naive CD4 T cells, B cells inhibit the capacity of DCs in producing Th1-associated IL-12 and thus indirectly promote IL-4-associated Th2 responses [79]. Therefore, B cells are essential at shaping the Th2 cell response via antigen presentation and the regulation of Th2-priming capacity of DCs.

The role of B cells as APCs in regulating Th1 cell responses is less profound than Th2 cell responses [75,77], suggesting unique costimulatory signals by B cells that specifically promote Th2 cell development. Multiple reports have demonstrated that costimulatory molecules such as CD80/86 [80,81], OX40L [82–84], and ICOSL [85,86] expressed by B cells are critical for Th2 response in various experimental models. For instance, the adoptive transfer of CD80- and 86-deficient B cells into *N*. *brasiliensis*-infected B cell-deficient mice failed to induce IL-4 production, resulting in impaired Th2 cell development without altering the Th1 cell response [80]. In the absence of CD28-derived signals in vitro, Th1 cell-associated IFN-γ production is unaffected while IL-4 is greatly diminished [81], suggesting that CD28–CD80/86 interactions are more critical for the development of Th2 than Th1 cells. However, contrasting reports also showed that Th2 cell responses can develop independently of CD80/86-derived signals during infection with *N*. *brasiliensis* [87] and *T*. *muris* [88], suggesting that there is a CD80/86-independent pathway for Th2 cell development. In such cases, CD80/86 signals provided by B cells might not be necessary for Th2 responses.

Despite costimulatory-derived signals by B cells seeming to favour Th2 cell development in helminth infection, effector B cells, termed as Be1, positively regulate Th1 cell differentiation via IFN-γ and tumor necrosis factor (TNF) production [89,90]. Alternatively, IL-4- and IL-13-producing effector B cells (Be2) play an important role in Th2 cell polarization [78,89,91]. However, Be2-mediated Th2 responses have been shown to be insufficient to establish immunity against *H*. *polygyrus* [76]. Even though conventional TNF production is required for optimal IL-12-induced Th1 cell development [92] and IFN-γ production by Th1 cells [93], it is also required for Th2-derived IL-13 production in response to *T*. *muris* [94]. These studies suggest TNF is neither a specific feature of Be1 nor Be2 cells; however, its production confers protection against helminths by regulating Th1 cell development and function, in addition to IL-13 production by Th2 cells.

### Antibody-independent functions of B cells: Maintenance of lymph node architecture

In addition to supporting Th2 cell responses through cytokines and costimulation, B cells are also important for remodelling the architecture of LNs. In contrast to Th1 cells [95,96], an initial encounter between DCs and naive CD4 T cells for Th2 cell differentiation specifically occurs at the border between T and B (T/B) cell (interfollicular) regions in the LNs [80,97]. Both Th2 cell-associated DCs and activated CD4 T cells are required to localise near the B cell follicles for Th2 cell development to take place by up-regulating their CXCR5 expression [98]. LT-expressing antigen-activated B cells are crucial for regulating the expression of the CXCR5 ligand CXCL13 by stromal-derived follicular dendritic cells (FDCs) via a positive feedback loop [99,100]. In the absence of LT production, activated Th2 cell precursors are unable to migrate to the interfollicular regions and thus disrupt optimal Th2 cell development [97]. In addition, the deletion of the receptor for LT, LTβ-receptor (LTβR), specifically on B cells (CD19-*cre* x LTβR*fl/fl*) fails to generate B cell follicles and the reorganization of FDCs around the B cell follicles, all of which is associated with impaired worm expulsion following primary infection of *H*. *polygyrus* [35]. More recently, it has also been shown that IL-4Rα-expressing B cells provide important LT-mediated interactions with CD19-expressing fibroblasts to regulate CXCL13 production by fibroblastic reticular cells within the interfollicular regions [101]. The loss of IL-4Rα expression exclusively by B cells in chimeric mice results in impaired protection against the parasites [101]. These studies suggest that the cross-talk between B cells and stromal cells is important for remodelling the architecture of the LNs, which is necessary for the development of protective immunity against helminths.

### IL-10-producing B regulatory (Breg) cells

Due to their immunomodulatory capacities, helminths have been harnessed as a potential therapy for a wide range of inflammatory diseases, as they can potently induce regulatory cell populations such as Treg cells. The abilities of Treg cells to suppress inflammation are primarily through their immunosuppressive cytokine production such as IL-10 and TFG-β [14]. Aside from Th1/Th2 cell-polarizing cytokines, B cells can also produce IL-10 and are referred to as regulatory B (Breg) cells. The roles of IL-10-producing Breg cells for protective responses have been extensively demonstrated during schistosomiasis, mainly due to their potent suppression of immunopathology associated with tissue damage [14,102]. Such suppression of immunopathology by Breg cells is via regulation of monocyte infiltration [103], as well as by controlling a balance between Th1 and Th2 cell responses [104,105]. During schistosomiasis, uncontrolled Th2 responses are responsible for damaging pathology [106]. As the absence of B cells promotes excessive Th2 polarization, the B cells are therefore deemed to be required for regulating schistosomiasis-associated, Th2-mediated immunopathology [106,107].

Although the roles of Bregs are primarily demonstrated in experimental schistosomiasis, mice lacking the B cells that fail to class-switch their IgM to any other isotypes and are unable to secrete antibodies (IgMi^−/−^ mice) have identified an interesting connection of Bregs to susceptibility and gut immunopathology following *T*. *muris* infection [108]. In contrast to schistosomiasis, the resistance to *T*. *muris* infection requires Th2-biased responses without the need for a balanced Th response between Th1 and Th2 components [109]. The susceptibility of IgMi^−/−^ mice towards a supposedly resolving acute infection is associated with IL-10 production by B cells during early stages of infection, with dysregulated Th2 responses at later time points of infection [108]. Conversely, in the chronic setting of *T*. *muris* infection, the intestinal crypts of IgMi^−/−^ mice had increased apoptotic cells, with elevated IL-10 production by B cells [108]. This indicates that antibodies but not B cell-derived IL-10 are in part responsible for worm clearance and maintaining gut homeostasis during acute and chronic *T*. *muris* infection, respectively. The ability of B cells to produce cytokines such as IL-10 demonstrates how regulatory functions of these cells could be an important component of protective responses. Clearly, their requirements through this pathway are context dependent and variable between different helminth infections (e.g., schistosomiasis versus *T*. *muris* infection).

## Antibody-mediated protective roles

There is conflicting evidence on the requirements of antibodies for protective responses to helminth infection; however, a small number of studies have demonstrated that antibodies are capable of protecting the host via direct interactions with invading parasites. Such direct interactions may hinder important aspects of the well-being of the parasites such as their growth, motility, tissue invasiveness, and reproductive fitness, depending on their stage of development and tissue niches. The basic life cycle of intestinal helminths typically starts with hatching of embryonated parasitic eggs into larvae 1 (L1), followed by 3 moulting stages (L1→L2→L3→L4) and an adult stage. The L3 and L4 moults, as well as adult worms are often associated with intestinal tissue residency. Depending on the stage of the infection, antibodies produced during the response could impair the parasites via direct interactions and/or signal the innate cells to promote worm expulsion.

Distinct tissue niches by different helminth species puts another layer of complexity on B cell regulation including GC reactions and antibodies and their roles during helminth infection. For example, infective L3 of *N*. *brasiliensis* from infected sites in skin first migrate to and localise in the mouse lungs for 18 to 72 hours, which then develop into L4 [110]. Given the right conditions that favour the parasite, the L4 can be transported out of the lungs to be ingested in the host intestine. Here, the L4 develops into an adult worm and stays in the intestine up to 7 days due to Th2 cell-dependent expulsion [110,111]. In contrast to *N*. *brasiliensis*, *T*. *muris*, *H*. *polygyrus*, *Strongyloides*, and *Ascaris* species do not localise in the lungs. Rather, the larvae directly migrate to and mature in the host intestine. The infectious cycle of *T*. *muris* inside the host starts with L1, whereas *H*. *polygyrus* and *N*. *brasiliensis* begin with infective L3 [109,110,112]. The expulsion of *T*. *muris* typically requires 21 days and is primarily mediated by type 2 responses which are also dependent on the numbers of infective eggs and mouse strain [109]. Like *T*. *muris*, *H*. *polygyrus* expulsion is also strongly influenced by mouse stains and its clearance may take 4 to 20 weeks [112]. The primary infection of *H*. *polygyrus* can be experimentally cleared using drugs such as ivermectin to promote rapid expulsion, which generates memory immunity to reinfection [112,113]. On the other hand, *Trichinella* species complete its life cycle inside the host and its infective muscle larvae matures to adult worms in the small intestine, where newborn larvae can be produced and expelled so that they can localise into skeletal muscle [114,115]. Altogether, it remains a challenging task to pinpoint the role of B cells, GC reactions, and antibodies during helminth infection, as the length of time that the parasite can remain in the host, as well as their favoured tissue niches, greatly vary amongst different helminth species.

### Direct effects of antibodies on the parasites

The most common experimental model used to study the roles of antibodies during helminth infection is passive immunization, which involves transfer of whole immune serum or purified antibodies into the host. The evidence for direct binding of antibodies to the parasites is rare and has been demonstrated in responses to only a handful of helminths such as *T*. *spiralis* [116,117], *Trichinella britovi* [118], *Strongyloides stercoralis* [119], and *Ascaris suum* [120]. During *T*. *britovi* infection, the antibodies bind to the stichocytes and cuticular parts of larvae but not the adults [118]. Importantly, antibody-dependent protection against *T*. *britovi* is only efficient in mice that received purified antibody before or immediately right after infection but not when the adult worms have fully matured [118], suggesting that anti-*T*. *britovi* antibodies are only effective against larval parasites. The direct interactions of antibodies with *T*. *spiralis* larvae prevents invasion of epithelial cells by specifically binding to glycans expressed on the surface of the larvae that is thought to be essential for the chemosensory reception of the parasites, particularly during the moulting stages [116,117]. More recent studies have also shown that the transfer of monoclonal antibodies reduces muscle larvae burden prior to migration to the intestinal tissue following rechallenge with *T*. *spiralis* [121,122]. These results collectively suggest that one of the direct protective effects of antibodies is by curtailing larval development and invasion of tissues, which provides early protection against helminth infection.

Unlike *T*. *britovi*, IgG1 antibodies do not mediate larval killing despite the binding of nonspecific, cross-reactive, or even parasite-specific IgG1 antibodies to the migrating larvae surface of *A*. *suum* in the lungs [120]. This finding is however contradicted with multiple transfers of immune IgG1 antibodies that have been shown to reduce parasite burden by possibly mediating larval killing of *A*. *suum* [123]. These studies raise an unresolved yet fundamental question on host–parasite interactions, and in cases where antibodies promote host immunity, how exactly the binding of antibodies to the parasite can impede its survival? Perhaps, there is also a requirement for certain levels of antibodies that need to bind to the larvae surface in order to promote larval killing, which remains to be tested.

### Antibodies predominantly mediate recall responses

Passive transfer of purified immune antibodies or/and sera induces protection against *A*. *suum* [123,124], *Ancylostoma caninum* [125], *T*. *spiralis* [116,121,122,126], *T*. *britovi* [119], *S*. *stercoralis* [119,127–129], *H*. *polygyrus* [76], and *T*. *muris* [17] (Table 1). The primary protection against *H*. *polygyrus* [130] and *S*. *stercoralis* [131] is independent of antibodies, suggesting that antibody requirements are more dominant during recall responses. A possible explanation for why antibodies induced during primary infection appear to be less protective might be that antibody response (IgG1 and IgE) is more robust (quantity) with increasing affinity and specificity for the antigen (quality) during recall responses [33,130]. Additionally, this could also be partly explained by the location of the parasite, where the access of antibodies to or antibody-mediated effects on adult worms residing in intestinal lumen could be hindered and less efficient during primary infection. In addition to passive immunization, a variety of murine models of B cell receptor (BCR) transgenic and gene targeted knockouts have significantly helped expand a current growing body of evidence on antibody roles during helminth infections. For example, mice lacking the heavy chain joining region in their BCR (J_H_D^−/−^ mice) to deplete functional B cells or lacking activation-induced cytidine deaminase (AID^−/−^ mice) to disable the abilities of their B cells to undergo isotype class switching or SHM, all fail to expel adult worms as efficiently as control mice following secondary response of *H*. *polygyrus* infection [33] (Table 2). By contrast, there is no effective immunity to primary *H*. *polygyrus* infection in C57BL/6 mice that can result in a sterile immunity [112], and thus the absence of B cells and antibodies does not influence the susceptibility; however, they can promote intestinal repair [132]. Conversely, μMT^−/−^ mice [17,74], B cell-depleted mice via anti-CD20 treatment [17,74], and mice that are unable to secrete any soluble antibodies (IgMi^−/−^ mice) [108] all fail to protect the host during primary *T*. *muris* infection (Table 2). Furthermore, J_H_D^−/−^ mice had impaired worm expulsion and increased fecal eggs in response to primary *Strongyloides venezuelensis* infection [133]. These studies highlight that the antibody-mediated protection against helminths during primary versus secondary responses is highly variable across different species of parasitic worms. It is likely that different species of helminths secrete unique excretory-secretory (ES) products that in turn indirectly influences the requirements of antibodies during primary versus secondary responses.

**Table 1:** Experiments using a passive immunization approach to study the role of antibodies during intestinal helminth infection.

Helminth species	Outcome	Proposed mechanism	Reference
*Ascaris suum*	Transfer of purified IgG1 and IgE induces more than 70% protection against the parasites.	Antibody-mediated reduction of parasite burden	[124]
*Trichuris muris*	Transfer of purified parasite-specific but not nonspecific IgG1 from resistant mice into mature B cell-deficient mice (μMT^−/−^ mice) significantly reduces worm burden.		[17]
*Strongyloides stercoralis*	Transfer of human immune serum or purified IgG induces larval killing in implanted diffusion chambers.		[119,127,128]
*Ascaris suum*	Multiple transfers of parasite-specific IgG derived from immunized mice reduces by at least 64% of larvae burden.		[123]
*Heligmosomoides polygyrus*	Transfer of parasite-specific IgG derived from vaccinated mice reduces adult worm burden.		[146]
	Transfer of parasite-specific IgG derived from vaccinated mice reduces parasitic egg output.	Antibody-mediated impact on worm fecundity	
	Transfer of naive serum or purified IgG into mice lacking a heavy chain joining region in their B cell receptor (J_H_D^−/−^ mice) reduces parasitic egg output.		[33]
*Nippostrongylus brasiliensis*	Transfer of immune serum into infected WT mice reduces parasitic egg output.		[130]
*Trichinella britovi*	Transfer of immune IgA renders protection in a dose-dependent manner.	Antibody-mediated reduction of parasite burden	[118]
*Ancylostoma caninum*	Transfer of serum from immune, vaccinated mice produces about 54%–63% reduction in larvae 3 in the lungs upon challenge.		[125]
*Heligmosomoides polygyrus*	Transfer of immune serum into J_H_D^−/−^ mice following secondary inoculation attenuates adult worm burden.		[130]
*Trichinella spiralis*	Transfer of parasite-specific serum reduces by at least 95% of muscle larvae development.		[126]
*Strongyloides stercoralis*	Transfer of immune serum into activation-induced cytidine deaminase-deficient mice (AID^−/−^ mice) restores worm expulsion.		[129]
*Trichinella spiralis*	Transfer of immune serum and eosinophils into eosinophil-deficient mice prevents migratory newborn larvae towards skeletal muscle upon reinfection.		[145]
	Transfer of 8F12 monoclonal antibodies targeting the parasite induces 24%–25% reduction in muscle larvae burden.		[121]
	Transfer of monoclonal antibodies against the complement C9 binding domain reduces larvae burden upon challenge.		[122]

**Table 2:** Experiments using specific mouse models to study the role of antibodies and B cells during intestinal helminth infection.

Type of B cell/antibody-targeted mouse model	Helminth species	Outcome	Proposed mechanism	Reference
Membrane-bound-IgM-deficient mice (μMT^−/−^)	*Trichuris muris*	μMT^−/−^ mice failed to expel adult worms following primary infection. Parasite antigen-restimulated mesenteric lymph node (MLN) cells produced naive levels of Th2 cytokines.	B cells mediate effector Th2 response during primary infection	[17]
	*Litomosoides sigmodontis*	Vaccinated μMT^−/−^ mice failed to produce degranulated eosinophils and harboured more larvae 3 than control mice.	Antibody-mediated eosinophil response	[134]
	*Strongyloides stercoralis*	Immunized μMT−/− mice exhibited 17% reduction in larvae recovery upon challenge.	B cells mediate larvae expulsion upon recall	[131]
	*Heligmosomoides polygyrus*	Infection-primed μMT^−/−^ mice harboured higher numbers of adult worms and failed to induce protective germinal center (GC) response and memory Th2 cell response.	GC B cells mediate memory Th2 response	[76]
		Vaccinated μMT^−/−^ mice failed to expel adult worms.	B cells mediate worm expulsion upon recall response	[146]
Mice lacking activation-induced cytidine deaminase-deficient (JHD^−/−^)		J_H_D^−/−^ mice harboured a higher worm burden upon secondary infection and more motile larvae burden than control.	Antibody-mediated reduction of parasite burden upon recall response	[33,130]
	*Strongyloides venezuelensis*	JHD^−/−^ exhibited susceptibility and increased fecal eggs.	B cells mediate worm expulsion	[133]
Activation-induced cytidine deaminase-deficient mice (AID^−/−^)	*Heligmosomoides polygyrus*	AID^−/−^ mice harboured more adult worms upon recall response than control and suffered impaired intestinal repair following primary infection.	Isotype-switched antibody is necessary for worm expulsion upon recall response and regulates intestinal repair	[33,132]
Mice that are unable to secrete any soluble antibodies (IgMi^−/−^)	*Trichuris muris*	IgMi^−/−^ mice exhibited higher numbers of adult worms and exhibited more inflamed intestinal tissue than control. Parasite antigen-restimulated MLN cells failed to produce IL-13.	Antibody-mediated worm expulsion during primary response	[108]
IgE-deficient mice (IgE^−/−^)	*Nippostrongylus brasiliensis*, *Trichinella spiralis*	IgE^−/−^ mice retain the ability to remain resistant to primary infection.	IgE-independent worm expulsion	[142]

### Antibody-mediated innate cell activity

To tailor the B cell response to produce antibody with the effector function most suited to the infecting pathogen, B cells undergo class switching from IgM to one of the downstream isotypes: IgG, IgA, or IgE, which depends on various factors such as the types of pathogens, gene transcription programs, and cytokines. Further, IgG has multiple subclasses, which have been differentially associated with Th2 versus Th1 responses. During Th2 cell-skewed responses, IL-4 production by activated Th2 cells is associated with IgE and IgG1 class switching. The proposed mode of how antibodies mediate protection is by activating innate cells such as eosinophils, macrophages, neutrophils, and mast cells at the effector site. Mice lacking antibodies failed to produce degranulated eosinophils and such a response is associated with impaired parasite clearance [134]. This is likely due to parasite-coated antibodies such as IgE that can potently bind Fc epsilon Receptor 1 (FcεR1)-bearing effector cells, which results in degranulation and the release of soluble antiparasitic mediators [10]. Alternatively, larval trapping is another host defence mechanism against tissue migrating larvae, which is facilitated by antibodies as mice lacking antibodies or Fc receptors harbour more motile larvae of *H*. *polygyrus* than control and thus suffer severely from parasite-associated tissue damage [135]. Mechanistically, *H*. *polygyrus*-specific IgG1 antibodies activate macrophages to directly trap larvae via CD64 complement receptor, resulting in larval immobilization, as well as promote intestinal repair [135,136]. Nevertheless, such an antibody-mediated larval trapping mechanism has only been demonstrated in *H*. *polygyrus*. Since IgG1 antibodies are also well associated with at least partial Th2-dependent protection against primary *T*. *muris* [17,137] and *T*. *spiralis* [121,138], it remains to be further investigated whether IgG1 antibodies can also employ the same larval trapping mechanism as seen in *H*. *polygyrus*.

Even though the protective roles of IgE in anti-helminth immunity are controversial (Table 2), IgE is thought to support the host immune response to clear the infection via innate cell activity. It is only recently demonstrated that IgE plays an essential yet opposing role in controlling the severity of *N*. *brasiliensis* infection, depending on the source of IgE [139]. B2 but not B1 cell-derived IgE reduces the levels of *N*. *brasiliensis* infection in a mucosal mast cell degranulation manner. However, the host-protective effects of B2 IgE are impeded in the presence of B1 IgE [139]. Mechanistically, blockade of B2 IgE-mediated mast cell degranulation is due to FcεR1 saturation by B1 IgE [139]. Clearly, the sources of IgE are an important factor for IgE-mediated mast cell activity during *N*. *brasiliensis* infection, although whether this holds to be true in other helminth models such as *T*. *muris* and *T*. *spiralis* is unknown. Ultimately, it still comes down to the requirement of IgE-mediated responses for anti-helminth immunity. In the case of IgE-independent protection mechanisms, the sources of IgE (e.g., B1 versus B2) could be of no significance in protective responses.

The roles for IgE during helminth infection in humans are still unclear due to a typical nature of polyparasitism among populations in endemic areas; however, serological studies in children and adults during trichuriasis [140] and ascariasis [141] suggest a clear negative correlation between parasite-specific IgE and infection intensity. Nevertheless, mice lacking IgE (SJA/9 mice) retain a similar ability as control mice to expel the worms following primary *N*. *brasiliensis* and *T*. *spiralis* [142] infection. This suggests there are other factors that could influence the antibody-independent innate cell activity against the helminths such as the immunomodulation by the ES products, tissue-derived alarmins, and commensal-derived molecules [143], which could be responsible for IgE-independent mast cell activation in promoting worm expulsion. The roles of protective antibodies are more apparent during a rechallenge response. Indeed, the secretion of antibody-mediated eosinophil-derived proteins impairs parasite integrity during secondary infection of *N*. *brasiliensis* [144] and *T*. *spiralis* [145], by reducing the migratory abilities of larvae to the lungs and intestinal tissue, respectively. A likely rationale for why the antibody-mediated anti-helminth innate cell activity seems to be important upon rechallenge but not during primary infection is that the rechallenge antibodies might possess a higher affinity at binding an epitope expressed on innate cells that are required for promoting worm expulsion.

### Antibody impact on worm fecundity

Protection against helminths is often associated with a significant reduction of worm burden. However, the term “protection against helminths” can also be associated with worm fecundity. For example, the transfer of immune serum into mice lacking B cells following *N*. *brasiliensis* infection does not promote worm expulsion [130]. However, the antibodies do impair the production of eggs and hence reduce the reproductive fitness of the worms [130,139]. A similar outcome of reduced parasitic egg output is also observed in response to *H*. *polygyrus*, as demonstrated by passive transfer of naive serum or IgG [33]. Following reinfection with *H*. *polygyrus*, polyclonal and parasite-specific IgG antibodies appear to have distinct yet complementary protective roles against the parasites [33]. For example, polyclonal IgG antibodies with irrelevant antigen specificities are required to limit parasite fecundity during early responses, while delayed antigen-specific IgG antibodies are to control adult worm growth [33]. However, the transfer of parasite-specific IgG antibodies could also reduce *H*. *polygyrus* fecundity, suggesting that antigen-specific antibodies may have both abilities at limiting egg production and worm growth [146]. Understanding how antibodies impact worm fecundity may provide an alternative approach for new interventions that specifically target worm reproduction, although this may be effective in only some helminth systems and such a defence mechanism by antibodies remains to be tested in other helminth infections other than *H*. *polygyrus*.

### Does microbiota-mediated antibody response promote or impede anti-helminth immunity?

As intestinal helminths share a niche with intestinal commensal microbes, the presence of helminths alters the composition of intestinal microbiota through their direct contact and the secretion of ES product, which influences the extent of host immunity to diseases such as allergies and inflammatory bowel disease [147,148]. Intuitively, the shaping of intestinal microbiota by helminths can favour both resistance and susceptibility; however, it still strongly relies on whether the changes in microbiota promote protective type 2 response or favour parasite survival. Recently, it is shown that specific pathogen-free and germ-free mice generate similar levels of Th2 responses, with effective worm expulsion of intestinal *Hymenolepis dimunita* [149]. This is observed despite the changes to the colonic microbiota after infection, indicating that protection against *H*. *diminuta* is independent of intestinal microbiota. In accordance with this finding, *S*. *mansoni*-infected mice after antibiotic treatment also displayed no changes in worm expulsion but had reduced intestinal tissue damage [150], suggesting that there are roles for microbiota at regulating immunopathology associated with the parasites. Intriguingly, despite no changes in early worm burden of *H*. *polygyrus* in germ-free mice (2 weeks postinfection), the worm size and egg production in these mice were reduced [151]. At later time points postinfection (4 to 6 weeks), germ-free infected mice harboured fewer adult *H*. *polygyrus* than control mice [152] and colonization with *Lactobacillus* correlates with infection intensity in C57BL/6 mice [153], suggesting that intestinal microbiota–parasite interaction and its correlation with protection against helminth changes throughout infection. While it is clear that IgA production by B cells is dependent on specific commensals that exist in the gut microbiota [154,155] and is important to provide partial protection against *T*. *muris* [156] and reinfections with *H*. *polygyrus* [33], the data to suggest what specific gut microbiota that mediate B cell and protective humoral responses against helminth are scant, and the cross-talk between helminths, intestinal microbiota, and the B cells, as well as antibodies, remains to be explored.

## Concluding remarks

The importance of B cells for protective immunity following helminth infection is clearly dependent on many factors such as the specific type of helminth parasites, developmental stages of the parasites, host genetic background, primary versus recall responses, and experimental settings. Although B cells do not provide total protection or sterilizing immunity in some helminth infections, their various roles are arguably necessary to afford some extent of anti-helminth type 2 immunity. The cellular mechanisms through which B cells could play an important role in protective responses include antibody production and regulation of the type 2 immunity through their interactions with Th cells, although these roles are varied across different intestinal helminth species. Furthermore, GC reactions may act as a reservoir for Th2-like Tfh cell response that in turn influences type 2 immunity. Antibodies that have been shown to contribute to protection in some helminth infection models are primarily demonstrated by passive transfer experiments. Several helminth systems elicit a strong type 2 immune response that is neither mediated nor dependent on antibodies and B cells. A key question that remains to be further explored is, how do the factors that determine the requirement of B cells and antibodies during helminth infections influence type 2 protective immunity? Although current deworming drugs are safe and relatively effective, they fail to provide long-term protection. Hence, continuing to understand the roles of B cells and their interactions with other cells during helminth infection is important for developing novel long-term interventions.
